# Remotely Assessing Motor Function and Activity of the Upper Extremity After Stroke: A Systematic Review of Validity and Clinical Utility of Tele-Assessments

**DOI:** 10.1177/02692155241258867

**Published:** 2024-06-05

**Authors:** Lena Sauerzopf, Andreas R. Luft, Anna Baldissera, Sara Frey, Verena Klamroth-Marganska, Martina R. Spiess

**Affiliations:** 1 30944 ZHAW School of Health Sciences, Institute of Occupational Therapy, Winterthur, Switzerland; 2 Faculty of Medicine, 27217 University of Zurich, Zurich, Switzerland; 3 Department of Neurology, Division of Vascular Neurology and Neurorehabilitation, 27217 University of Zurich, Zürich, Switzerland; 4 Care Home City of Winterthur, Winterthur, Switzerland; 5 Adelheid Clinic, Rehabilitation Center Central Switzerland, Unterägeri, Switzerland

**Keywords:** Tele-assessment, review, motor function, stroke

## Abstract

**Objective:**

The aim of this systematic review is to identify currently available tele-assessments for motor impairments of the upper extremity in adults after a stroke and to assess their psychometric properties and clinical utility.

**Data sources:**

We searched for studies describing the psychometric properties of tele-assessments for the motor function of the upper extremity. A systematic search was conducted in the Cumulative Index to Nursing and Allied Health Literature, Medline via OVID, Embase, The Cochrane Library, Scopus, Web of Science and Institute of Electrical and Electronics Engineers Xplore from inception until 30 April 2024.

**Review methods:**

The quality assessment for the included studies and the rating of the psychometric properties were performed using the COSMIN Risk of Bias Checklist for systematic reviews of patient-reported outcome measures.

**Results:**

A total of 12 studies (N = 3912) describing 11 tele-assessments met the predefined inclusion criteria. The included assessments were heterogeneous in terms of quality and psychometric properties and risk of bias. None of the tele-assessments currently meets the criteria of clinical utility to be recommended for clinical practice without restriction.

**Conclusion:**

The quality and clinical utility of tele-assessments varied widely, suggesting a cautious consideration for immediate clinical practice application. There is potential for tele-assessments in clinical practice, but the clinical benefits need to be improved by simplifying the complexity of tele-assessments.

**Registration Number:**

CRD42022335035.

## Introduction

In mitigating the escalating costs of stroke rehabilitation, the trend has shifted from extended inpatient care to early discharge, emphasizing outpatient and home-based therapy.^1^ Within this context, and accelerated by the COVID-19 pandemic, tele-rehabilitation (i.e., providing rehabilitative treatments “at a distance” between the clinician and the patient's home) has gained significant interest.^2,3^

Tele-rehabilitation recommendations emphasise the relevance of therapist monitoring and adjusting interventions based on progress towards established goals.^4^ Reliable and valid measurements for assessing the motor function following stroke are, therefore, essential.^5^ Tele-assessments like in-person evaluations must exhibit reliability, validity and sensitivity to change.^6^

Previous work in post-stroke motor rehabilitation has focused on assessments provided in person (whether technology-supported or not)^7–10^ and interventions delivered remotely.^11–13^ Whilst adapting questionnaires for remote use appears straightforward^14^; “hands-on” assessments present greater challenges in remote settings. As of now, there is no comprehensive overview of such “hands-on” assessments for gauging the motor function and activity after stroke in remote contexts.

This review aims to identify clinically ready-to-use tele-assessments for the evaluation of the motor function and activity of the upper extremity post-stroke and to systematically investigate their measurement properties. Additionally, the clinical utility of these tele-assessments will be evaluated, and recommendations for their use will be provided.

## Methods

This systematic review adheres to the “reporting guideline for systematic reviews of outcome measurement instruments” (PRISMA-COSMIN for OMIs, currently in development)^15^ and is registered in PROSPERO (CRD42022335035). The qualities of the studies and the tele-assessments of the included papers are assessed separately. When necessary, additional data or clarifications were requested from the authors.

Studies were included if they met the following criteria: they tested a tele-assessment in adults (≥18) post-stroke; they reported about a tele-assessment, which measures the motor function or activity of the upper extremity and was either developed for remote use or adapted from a traditional in-person assessment for remote use; the tele-assessment measures the movement quality (i.e., how the movement was executed) rather than just quantity; the tele-assessment is clinically ready to use (i.e., completed remote testing, either between research facility and participants’ homes or between two separate rooms in a research facility); and the study was published in a peer-reviewed, scientific journal. Moreover, studies that solely provide information on patients’ or therapists’ perspectives or opinions towards tele-assessments and reviews were excluded.

### Searches and Data Sources

A systematic literature search was initially conducted in July 2022 and last updated on 30 April 2024. Given the interdisciplinary nature of this topic, we incorporated the following seven databases: Cumulative Index to Nursing and Allied Health Literature (CINAHL), Medline via OVID, Embase, The Cochrane Library, Scopus, Web of Science and IEEE Xplore. The search strings were built using the Boolean operator OR between synonyms and the Boolean operator AND between concepts (“stroke”, “sensorimotor”, “teleassessment” and “upper extremity”). Controlled vocabulary and indexing systems as well as free text words were used for the search. The search strings were adapted to the respective databases. All search strings were developed in consultation with a librarian at the University of Zurich in April 2022. We included studies in all languages and of all publication dates. We translated two studies that were only available in Chinese using DeepL to be able to appraise the full texts. A full-search strategy for the CINAHL can be found in Supplement Table 1. The search strategies of the other databases are available on request from the authors.

All initially identified hits were imported into “Covidence”.^16^ Duplicates were removed automatically and manually. Four reviewers screened titles and abstracts for eligibility, with each abstract being reviewed by at least two of these reviewers. Conflicts were resolved by an independent third reviewer. The remaining articles were subjected to full-text screening, whereby each article was evaluated independently by two of the same reviewers. Conflicts were resolved in team discussion.

The first author conducted data extraction for the included studies. For quality assurance, a second reviewer independently extracted a random data sample for comparison. A structured form (see online Supplementary Table 2) was used to extract the study characteristics (e.g. evidence level), characteristics of the tele-assessments (e.g. description of the assessment, psychometric properties) and results (e.g. usability the described tele-assessment).

### Study Quality Assessment

We assessed the risk of bias (methodological quality) of the included studies using an adapted version of the COSMIN Risk of Bias Checklist for systematic reviews of patient-reported outcome measures.^17^ The psychometric properties of reliability (COSMIN Box 6), measurement error (COSMIN Box 7), criterion validity (COSMIN Box 8), hypotheses testing for construct validity–convergent validity (COSMIN Box 9a) and construct approach (COSMIN Box 10b) of the included studies were analysed by two reviewers independently. Each criterion's (box's) score was determined by the lowest value among the respective items.^18^ In addition, we applied the criteria for good measurement properties on a three-point rating scale (sufficient, insufficient and indeterminate).^19^ Discrepancies were resolved with discussion. The criteria for good measurement properties used in this systematic review are listed in Supplement Table 4.

### Clinical Utility

We used two tools to critically appraise the clinical utility of the tele-assessments. First, the overall clinical usefulness according to Fawcett^20^ was chosen for its systematic approach to provide an in-depth qualitative overview. Here, clinical utility is described by the factors of cost, time, energy and effort, portability and acceptability. Second, the quantitative 10-point clinical utility framework by Tyson and Connell^21^ was applied. Assessments scoring 9 or 10 are recommended for clinical use.^21^

## Results

**Figure 1.**
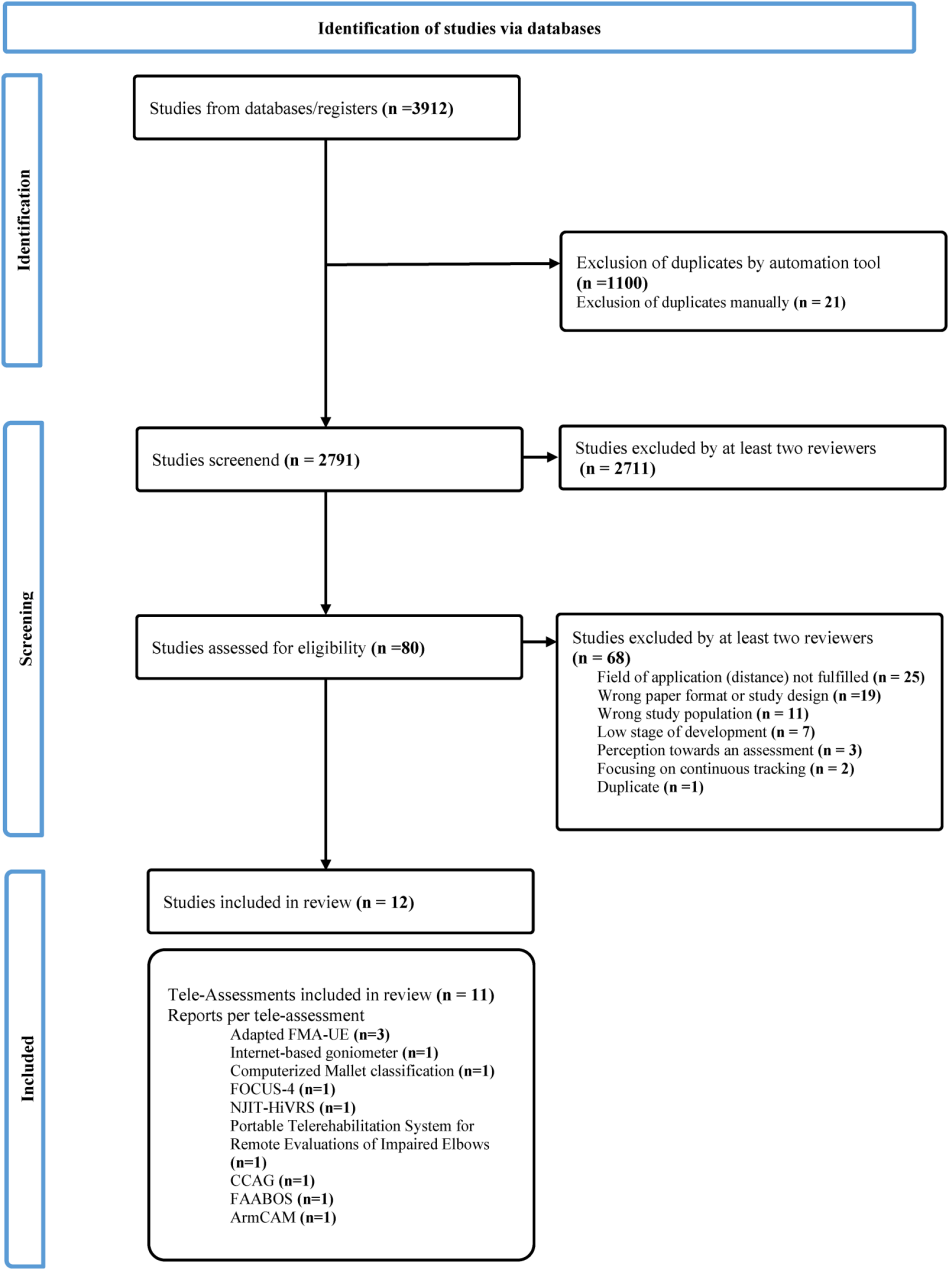
Preferred reporting items for systematic and meta-analysis (PRISMA) flow chart.

The search resulted in 3912 publications, of which 80 were evaluated in full text, and 12 were included for data extraction (Figure 1).^22–33^ These 12 studies described 11 different (versions of) tele-assessments measuring the motor function or activity. Thirty-two studies were excluded during full text screening either because they had not been tested at a distance^34–57^ or were generally still in a very early stage of development.^58–64^ Another study did not fully meet the inclusion criteria.^53^ We mention this special case because the authors have evaluated usability, but not specifically the psychometric properties of the assessment at a distance.

The characteristics of the 12 included studies are presented in Table 1. Six out of the 11 tele-assessments investigated modifications of existing clinical assessments, and five described assessments specifically developed for remote use (Table 1). Sample sizes in the 12 studies ranged from four to 56 individuals. All studies enrolled participants who were more than three months post-stroke.

**Table 1.** Study characteristics.

Adaption of tele-assessments for use at a distance													
Instrument name	N (f/m)	Stroke type	Time since stroke/ recovery phase	Method of measurement	Method of classification	Number of subscales/ items^a^/measurements^b^	Max score	Response options	Reference, country	Setting	Type	Need for assistance	Standard clinical assessment
tUEFMA	22 (5/17)	NR	>12 months	Human visual observation	Manual/human-based	22 items (five subscales)	44	Three-point ordinal scale	Carmona et al. (2023), USA	Remotely (NR)	FO	Yes	FMA-UE
Quantitative Fugl–Meyer assessment framework	24 (8/16)	Cerebral haemorrhage (7); cerebral infarction (17)	4–12 months	Flex sensors, accelerometer sensors	Automated	Seven items	NR	NR	Yu et al. (2016), China	From lab to home	FO	No	FMA-UE
Tele-FMA	11 (1/10) 34 (9/25)	ischaemic stroke 64% (in-person and Tele-FMA) and 73% (Tele-FMA)	Subacute and chronic	Human visual observation	Manual/human-based	30 items^1^ (seven subscales)	60^1^	Three-point ordinal scale	Liz et al. (2023), Brazil	From lab to home	FO	Yes	Braztel-MEEM, Tele-FMA
Internet-based goniometer	10 (2/8)	NR	NR	Digital goniometer	N/A	Seven measurements (no subscales)	N/A	Degree	Hoffmann et al. (2007), Australia	Separate rooms in the lab	FO	Yes	Universal goniometer
Computerised Mallet classification^a^	7 (NR)	NR	5–58 months	Kinect sensor	Automated	Five items (One scale)	15	Three-point ordinal scale	Seo et al. (2019), USA	Remotely (videotapes)	AO	NR	NR
FOCUS-4	26 (9/17) 27 (9/18) 56 (24/34)	NR	3 months to chronic stage	Human visual observation	Manual/human-based	Three items	57	Three-point ordinal scale	Jordan and Stinear (2024), New Zealand	Separate rooms	FO	Yes	ARAT
**Development of tele-assessments for use at a distance**													
**Instrument name**	**N (f/m)**	**Stroke type**	**Time since stroke/** **recovery phase**	**Method of measurement**	**Method of classification**	**Number of subscales/items/measurements**	**Max score**	**Response options**	**Reference, country**	**Setting**	**Type**	**Need for assistance**	**Standard clinical assessment**
NJIT-HiVRS	7 (2/5)	NR	7–53 months	Kinematic, Leap Motion Controller	N/A	Six measurements (no subscales)	N/A	N/A	MontJohnson et al. (2023), USA	Separate rooms in the lab	FO	No	NR
Portable Telerehabilitation System for Remote Evaluations of Impaired Elbows	4 (NR)	NR	7.8 ± 3.8 years	Telerobotic	N/A	Four measurements (no subscales)	N/A	Degree	Park et al. (2007, 2008), USA	Remotely (NR)	FO	Yes	NR
CCAG	33 (10/23)	NR	3–60 weeks	Kinematic, wireless controller	N/A	40 measurements (no subscales)	N/A	N/A	Serradilla et al. (2014), UK	From lab to home	FO	No	CAHAI-9
FAABOS	9 (2/7) 9 (4/5)	NR	4.1 years (1.9)	Accelerometer sensors	NR	N/A	N/A	Four-point ordinal scale	Uswatte and Hobbs Qadri (2009), USA	Remotely (Videotapes)	FO/AO	NR	NR
ArmCAM	31 (11/20)	NR	>6 months	Human visual observation	Manual/human-based	10 items (five subscales)	30	Four-point ordinal scale	Yang et al. (2023), Taiwan/Canada	From lab to home	FO/AO	Yes	SIS-Hand REACH FMA-UE ARAT

AO: activity-oriented; ARAT: Action Research Arm Test; ArmCAM: Arm Capacity and Movement Test; Braztel-MEEM: Brazilian Telephone Mini-Mental State Examination; CAHAI-9: Chedoke Arm and Hand Activity Inventory-9; CCAG: Circus Challenge Assessment Game; FAABOS: Functional Arm Activity Observation System; FO: function-oriented; FOCUS-4: Fast Outcome Categorization of the Upper Limb after Stroke-4; N/A: not applicable; NJIT-HiVRS: Kinematic Measures of Wrist and Finger Function by New Jersey Institute of Technology—Home Virtual Rehabilitation System; NR: not reported; REACH: Rating of Everyday Arm-Use in the Community and Home; SIS-Hand: Stroke Impact Scale — Hand; Tele-FMA: Tele-Fugl–Meyer Assessment for the Upper Extremity (excluding lower extremity); tUEFMA: Upper Extremity Fugl–Meyer Assessments for Telerehabilitation; UEFMA/FMA-UE: Upper Extremity Fugl–Meyer Assessment.

a Subscales and items are of a total score and meant to be summarised in a total score; ^b^Measurements are taken individually and are not meant to be summarised in a total score.

1 Tele-Fugl–Meyer Assessment for the Upper Extremity (excluding lower extremity).

Four tele-assessments were tested between the research facility and the participants’ homes and two in a research facility between separate rooms. Two studies validated movement scoring from video recordings by comparing them to in-person scoring. Authors of three studies did not provide further detail about the setup of remote testing (upon request; Table 1). The Fugl–Meyer assessment (FMA; *n *= 3), range of motion measurement using a goniometer (*n *= 1), Mallet classification (*n *= 1) and Action Research Arm Test (ARAT; *n *= 1) are established clinical assessments that were adapted for use at a distance.^22–26,33^

According to the respective authors, the quantitative Fugl–Meyer assessment framework,^23^ Kinematic Measures of Wrist and Finger Function by the New Jersey Institute of Technology — Home Virtual Rehabilitation System (NJIT-HiVRS)^27^ and Circus Challenge Assessment Game (CCAG)^30^ were designed, such that people after a stroke can set them up without assistance. For other included tele-assessments, it was explicitly stated that they were conducted with assistance involving clinicians at a distance or caregivers during the process (Table 1).^22,24,25,29,32,33^

### Quality Assessment

The results of the quality assessment and detailed information on the psychometric properties of the included tele-assessments are reported in Supplement Tables 3 and 5. Seven tele-assessments had at least one measurement property classified as “sufficient” in a study with the methodological quality determined as at least “adequate”.^22–25,30–32^

Reliability was assessed for nine of the 11 included tele-assessments.^22–27,31–33^ Sufficient reliability, a high-evidence level, was found for the Tele-FMA,^24^ tUEFMA (low-evidence level), Fast Outcome Categorization of the Upper Limb after Stroke-4 (FOCUS-4; low-evidence level), internet-based goniometer (high-evidence level), Functional Arm Activity Observation System (FAABOS; high-evidence level) and Arm Capacity and Movement Test (ArmCAM; high-evidence level).^22,25,31–33^

The measurement error was evaluated for six of the 11 included tele-assessments.^22,24–26,31,32^ It was deemed sufficient for Tele-FMA (high-evidence level).^24^ For the ArmCAM (high-evidence level),^32^ the measurement error was rated indeterminate for four tele-assessments.^22,25,26,31^

The criterion validity was assessed for five of the 11 tele-assessments.^22,24,27,30,32^ This item was rated sufficient for the tUEFMA (high-evidence level), Tele-FMA (high-evidence level), Circus Challenge Assessment Game (high-evidence level) and ArmCAM (high-evidence level).^22,24,30,32^

The established gold standard of the comparison measurement for the tUEFMA, the Tele-FMA and the ArmCAM was the FMA-UE, whilst for the Circus Challenge Assessment Game, the Chedoke Arm and Hand Assessment Inventory (CAHAI). For the Tele-FMA, the Stroke Impact Scale for Hand was additionally used as the gold standard for comparison. ArmCAM was additionally compared with the Stroke Impact Scale for Hand, the Rating of Everyday Arm-Use in the Community and Home (REACH scale) and the Action Research Arm Test (ARAT) for a subgroup. The criterion validity for the NJIT-HiVRS was rated insufficient because some measurements were below 0.70.^27^ An overview of the psychometric properties of the included tele-assessments is provided in Table 2.

**Table 2.** Psychometric properties of the included tele-assessments.

Adaption of existing clinical assessments for use at a distance						
Instrument name	Validity			Measurement Error	Reliability	
	*Convergent validity*	*Criterion validity*	*Construct validity*		*Intrarater reliability*	*Interrater reliability*
tUEFMA^22^		✓		•		•
quantitative FMA framework^23^	✓		•			•
Tele-FMA^24^	✓	✓	✓	✓	✓	✓
Internet-based goniometer^25^	•		•	•	✓	✓
Computerised Mallet classification ^26^	•			•		•
FOCUS-4^33^					•	
Development of tele-assessments for use at a distance						
Instrument name	Validity			Measurement Error	Reliability	
	*Convergent validity*	*Criterion validity*	*Construct validity*		*Intrarater reliability*	*Interrater reliability*
NJIT-HiVRS^27^	•	•			•	
Portable Telerehabilitation System for Remote Evaluations of Impaired Elbows^28,29^			•			
CCAG^30^	•	✓	•			
FAABOS^31^	•		•	•		✓
ArmCAM^32^	•	✓	•	✓	✓	✓

The checkmarks (✓) indicate that the measurement property was rated “very good” or “adequate” in terms of the risk of bias and additionally show sufficient summarised evidence.

The dots (•) indicate that the corresponding measurement property was assessed in the study, but shows an increased risk of bias and/or an inadequate or indeterminate summarised evidence.

ArmCAM: Arm Capacity and Movement Test; CCAG: Circus Challenge Assessment Game; FAABOS: Functional Arm Activity Observation System; FOCUS-4: Fast Outcome Categorization of the Upper Limb after Stroke-4; NJIT-HiVRS: Kinematic Measures of Wrist and Finger Function by New Jersey Institute of Technology—Home Virtual Rehabilitation System; Tele-FMA: Tele-Fugl–Meyer Assessment for the Upper Extremity (excluding lower extremity); tUEFMA: Upper Extremity Fugl–Meyer Assessments for telerehabilitation.

Supplement Table 5 shows an overview of the evidence of hypothesis testing for construct validity and responsiveness.

### Clinical Utility of Tele-Assessments

We describe the clinical utility according to Fawcett.^20^ All studies reported what additional technical equipment was required. In addition to commercially available hardware (e.g. phone, computer, off-the-shelf cameras and Kinect camera) and software (e.g. video conferencing programs), several of the tele-assessments require further equipment such as accelerometer sensors, telerehabilitation systems, or a Leap Motion Controller (LMC, UltraLeap, California, USA).^23,25–30^ The authors of three studies reported that their tele-assessment is cost-effective because of the low-cost equipment.^22,26,30^ One research group deals with the high cost of their tele-assessment by offering leasing options.^29^ Time to administer is outlined in four studies, varying between 5 min and approximately 20 min.^24,30,32,33^ Information on training effort and assistance (item Energy and Effort^20^) is given for nine out of 11 tele-assessments.^22–26,29,30,32,33^ All selected studies previously examined use at a distance (either home setting, room to room or otherwise remotely); hence, we assumed that all included tele-assessments are portable. In one study, the System Usability Scale was used to draw conclusions about usability and acceptability.^27^ In two other studies, it was reported that only some of the participants were satisfied with the home setting requirements.^23,24^ However, in one of these two studies, acceptance was reported by almost half of the participants (41%).^24^ Further details on clinical utility are shown in Supplement Table 6. Based on Tyson and Connell,^21^ none of the tele-assessments met the criteria for sufficient clinical utility (Table 3). We requested missing information from the authors, and in cases of a non-response, we made estimates using available evidence and clinical expertise. For the cost calculations, common household technologies (e.g. laptop and smartphone) were assumed and excluded from the calculation.

**Table 3.** Clinical utility of the selected tele-assessments.

Tele-assessment	Time to complete	Costs^e^	Portability^f^	Specialist equipment (and training)^g^	Total (max score: 10)
tUEFMA	2^a^	3	2	1	8
Quantitative FMA framework	2^a^	2	2	0	6
Tele-FMA	2	3	2	1	8
Internet-based goniometer	2^b^	2	2	0	6
Computerised Mallet classification	2^c^	2	2	0	6
FOCUS-4	3	3	2	0	8
NJIT-HiVRS	1^d^	2	2	0	5
Portable Telerehabilitation System for Remote Evaluations of Impaired Elbows	1^d^	1	1	0	3
CCAG	2	2	2	0	6
FAABOS	1^d^	2	2	0	5
ArmCAM	2	3	2	1	8

a The duration is not described in these studies. The authors assumed that the time to complete, according to the empirical values of the FMA-UE conducted in person, does not exceed 30 min.

b The duration is not described in these studies. The authors assumed that the time to complete, according to the empirical values of the goniometer conducted in person, does not exceed 30 min.

c The duration is not described in these studies. The authors assumed that the time to complete, according to the empirical values of the Mallet conducted in person, does not exceed 30 min.

d The duration is not described in these studies. However, based on the description in the studies, these values were assumed.

e None of the included studies reported the cost of the assessment. Costs were estimated by the authors based on the equipment specified.

f Portability was estimated by the authors based on the equipment specified.

g Specialist equipment: this item was also estimated based on the information in the studies.

ArmCAM: Arm Capacity and Movement Test; CCAG: Circus Challenge Assessment Game; FAABOS: Functional Arm Activity Observation System; FOCUS-4: Fast Outcome Categorization of the Upper Limb after Stroke-4; NJIT-HiVRS: Kinematic Measures of Wrist and Finger Function by New Jersey Institute of Technology—Home Virtual Rehabilitation System; Tele-FMA: Tele-Fugl–Meyer Assessment for the Upper Extremity (excluding Lower Extremity); tUEFMA: Upper Extremity Fugl–Meyer Assessments for telerehabilitation.

Description of the number system used^21^:

This scale evaluates the practical details of employing an assessment in clinical practice. Combining the scores results in a maximum total score of 10. A score of 9 or higher is necessary for the assessment to be considered recommended for clinical use.^21^

• Time to complete: for durations of less than 10 min, a score of 3 is assigned; durations between 10 and 30 min receive a score of 2; durations ranging from 30 to 60 min are given a score of 1; and durations exceeding 1 h are assigned a score of 0.^21^

• Cost: costs below £100 score 3; costs between £100 and £500 score 2; costs between £500 and £1.000 score 1; and costs above £1.000 or unknown costs score 0.^21^

• Portability: this question aims to determine whether the assessment is portable and whether it can be taken to the patient. “Yes, easily, can go in pocket” scores 2. “Yes, in briefcase or trolley” scores 1. “No or very difficult” scores 0.^21^

• Specialist equipment: this item addresses the question of whether or not the assessment requires specialist equipment and training for use. “No” scores 2. “Yes, but only simple, easy-to-use equipment which does not need specialist training to use” scores 1. “Yes” or “Unknown” scores 0.^21^

## Discussion

To our knowledge, this is the first review to describe and evaluate the measurement properties and clinical utility of the remote tele-assessments of the motor function and activity after stroke. We identified 11 tele-assessments in 12 studies that were examined for remote use (Table 1). The Mallet classification adapted for remote use was conducted in the study with post-stroke patients. It is important to emphasise that this classification for assessing brachial plexus palsy lacks prior validation for post-stroke use.^26^ The quality of the tele-assessments in terms of the measurement properties varied from “sufficient” to “insufficient” and methodological quality from “inadequate” to “very good”. The included studies showed a high degree of heterogeneity both in terms of the assessments themselves and the methods conducted for data collection and interpretation. This underlines the wide range of quality of the tele-assessments included. This finding also precluded a meaningful comparison between the tele-assessments, making it difficult to draw overarching conclusions regarding the overall validity and reliability of tele-assessments. Such conclusions must be drawn on an individual basis.

Prior reviews focused on a distinct category of measuring the motor function, for example, wearable devices like sensors.^65,66^ In addition, clinical utility was not addressed in these reviews. Appropriate clinical utility is an important key element for implementation in clinical practice, which must be considered in the development and contribution of tele-assessments.^67^ Whilst some of the tele-assessments we included demonstrated strong utility scores, none met the critical threshold of nine points, limiting our ability to make unreserved recommendations for clinical practice. Key limiting factors are technical considerations and/or training.^67^ All included tele-assessments require special equipment and/or training. In our context, this implies the following example: specialised tele-rehabilitation interfaces are needed,^29,30^ or training of caregivers is necessary to assist patients post-stroke in conducting the assessment.^24,33^ One added value of tele-rehabilitation is that it should save costs in the healthcare system. To minimise entry barriers for the implementation of tele-assessments and ensure the ongoing use of tele-rehabilitation, we recommend ensuring that the necessary equipment and training and general costs are kept at a minimum.

In the process, we found 32 tele-assessments that are at an early stage of development.^34–64^ These technologies have not yet undergone comprehensive testing for the use at a distance and did, therefore, not fulfil the inclusion criteria for this review. However, they show great potential in attaining clinical relevance with further refinement soon. They underline the dynamic and rapidly evolving nature of the domain of tele-assessments.

Two of these studies focused on establishing a ground truth for artificial intelligence (AI) applications in the assessment domain.^62,63^ Whilst we did not specifically search for AI-based assessments, we came across studies establishing a ground truth for AI applications during the whole literature search and selection process. We, therefore, anticipate that remote assessments will increasingly incorporate the AI technology in the future.

We acknowledge several limitations in this review. The findings of this review were constrained by incomplete reporting in the studies we included. Despite our efforts to request missing information from the authors, the responses were often missing. Therefore, we estimated certain values when assessing the clinical utility. The utility scale refers to the costs in British Pounds. We see limitations in the generalisability of the results to other regions with differing socio-economic standards. Another essential consideration about clinical utility is whether or not the devices hold the necessary certification. This aspect is not mentioned in the clinical utility scale and is outside the scope of this review, but should be considered in future work on this topic. In addition, studies were excluded from this review if there were uncertainties regarding testing at a distance, and the relevant information could not be obtained from the corresponding authors. This may have led us to exclude relevant studies at an early stage, which may have had an impact on the analysis.

In conclusion, this systematic review provides an overview of the emerging field of tele-assessments. We identified 11 different tele-assessments in 12 studies. The assessment and study quality varied widely, ranging from poor to very good. Reducing complexity, including materials and training, and lowering costs are desirable ways to improve.

**Clinical messages**

- None of the tele-assessments met the criteria for clinical utility, underscoring the need for cautious consideration in recommending their immediate adoption in clinical practice due to existing limitations.
- There is potential for responsible use, although we recognise that their clinical utility needs to be further improved.

## Supplemental Material

sj-docx-1-cre-10.1177_02692155241258867 - Supplemental material for Remotely Assessing Motor Function and Activity of the Upper Extremity After Stroke: A Systematic Review of Validity and Clinical Utility of Tele-Assessments Supplemental material, sj-docx-1-cre-10.1177_02692155241258867 for Remotely Assessing Motor Function and Activity of the Upper Extremity After Stroke: A Systematic Review of Validity and Clinical Utility of Tele-Assessments by Lena Sauerzopf, Andreas R. Luft, Anna Baldissera, Sara Frey, Verena Klamroth-Marganska and Martina R. Spiess in Clinical Rehabilitation

sj-pdf-2-cre-10.1177_02692155241258867 - Supplemental material for Remotely Assessing Motor Function and Activity of the Upper Extremity After Stroke: A Systematic Review of Validity and Clinical Utility of Tele-Assessments Supplemental material, sj-pdf-2-cre-10.1177_02692155241258867 for Remotely Assessing Motor Function and Activity of the Upper Extremity After Stroke: A Systematic Review of Validity and Clinical Utility of Tele-Assessments by Lena Sauerzopf, Andreas R. Luft, Anna Baldissera, Sara Frey, Verena Klamroth-Marganska and Martina R. Spiess in Clinical Rehabilitation

## References

[1] Federal Office of Public Health FOPH. Konzept Monitoring «Ambulant vor Stationär», https://www.bag.admin.ch/bag/de/home/versicherungen/krankenversicherung/krankenversicherung-leistungen-tarife/Aerztliche-Leistungen-in-der-Krankenversicherung/ambulant-vor-stationaer.html (2023, accessed 10 October 2023).

[2] Klamroth V Gemperle M Ballmer T , et al. *Does Therapy Always Need Touch? A cross-sectional study among Switzerland-based occupational therapists and midwives regarding their experience with health care at a distance during the COVID-19 pandemic in Spring 2020*. Preprint, In Review. Epub ahead of print 2021. DOI: 10.21203/rs.3.rs-103168/v1. 10.1186/s12913-021-06527-9 PMC8205206 34130691

[3] Dahl-Popolizio S Carpenter H Coronado M , et al. Telehealth for the provision of occupational therapy: reflections on experiences during the COVID-19 pandemic. Int J Telerehab 2020; 12: 77–92. 10.5195/ijt.2020.6328 PMC7757642 33520097

[4] National Clinical Guideline for Stroke for the United Kingdom and Ireland, www.strokeguideline.org. (2023). 10.1177/03080226231188020 PMC12033436 40337193

[5] Heran M Lindsay P Gubitz G , et al. Canadian stroke best practice recommendations: acute stroke management, 7^th^ Edition Practice Guidelines Update, 2022. Can J Neurol Sci 2022; 51: 1–31. 36529857 10.1017/cjn.2022.344

[6] WFOT WF of OT. Position Statement Occupational Therapy and Telehealth, https://www.wfot.org/resources/occupational-therapy-and-telehealth (2021, accessed 30 May 2021).

[7] Schwarz A Kanzler CM Lambercy O , et al. Systematic review on kinematic assessments of upper limb movements after stroke. Stroke 2019; 50: 718–727. 30776997 10.1161/STROKEAHA.118.023531

[8] Mesquita IA Fonseca PFPD Pinheiro ARV , et al. Methodological considerations for kinematic analysis of upper limbs in healthy and poststroke adults part II: a systematic review of motion capture systems and kinematic metrics. Top Stroke Rehabil 2019; 26: 464–472. 31064281 10.1080/10749357.2019.1611221

[9] Giarmatzis G Fotiadou S Giannakou E , et al. Understanding post-stroke movement by means of motion capture and musculoskeletal modeling: a scoping review of methods and practices. BioMed 2022; 2: 409–421.

[10] Hughes A-M Bouças SB Burridge JH , et al. Evaluation of upper extremity neurorehabilitation using technology: a European Delphi consensus study within the EU COST action network on robotics for neurorehabilitation. J NeuroEngineering Rehabil 2016; 13: 86. 10.1186/s12984-016-0192-z PMC5035444 27663356

[11] Sarfo FS Ulasavets U Opare-Sem OK , et al. Tele-rehabilitation after stroke: an updated systematic review of the literature. J Stroke Cerebrovasc Dis 2018; 27: 2306–2318. 29880211 10.1016/j.jstrokecerebrovasdis.2018.05.013 PMC6087671

[12] Sharififar S Ghasemi H Geis C , et al. Telerehabilitation service impact on physical function and adherence compared to face-to-face rehabilitation in patients with stroke: a systematic review and meta-analysis. PM&R 2023; 15: 1654–1672. 37139741 10.1002/pmrj.12988

[13] Laver KE Adey-Wakeling Z Crotty M , et al. Telerehabilitation services for stroke. Cochrane Database Syst Rev 2020; 1: 1–81. 10.1002/14651858.CD010255.pub3 PMC6992923 32002991

[14] Brito SAFD Scianni AA Peniche PDC , et al. Measurement properties of outcome measures used in neurological telerehabilitation: a systematic review using COSMIN checklist. Clin Rehabil 2023; 37: 415–435. 36448251 10.1177/02692155221129834

[15] Elsman EBM Butcher NJ Mokkink LB , et al. Study protocol for developing, piloting and disseminating the PRISMA-COSMIN guideline: a new reporting guideline for systematic reviews of outcome measurement instruments. Syst Rev 2022; 11: 121. 35698213 10.1186/s13643-022-01994-5 PMC9195229

[16] Veritas Health Innovation. Covidence systematic review software, www.covidence.org (2023).

[17] Mokkink L de Vet H Terwee C , et al. COSMIN Risk of Bias tool to assess the quality of studies on reliability and measurement error of outcome measurement instrument. 18. 10.1186/s12874-020-01179-5 PMC7712525 33267819

[18] Terwee CB Mokkink LB Knol DL , et al. Rating the methodological quality in systematic reviews of studies on measurement properties: a scoring system for the COSMIN checklist. Qual Life Res 2012; 21: 651–657. 21732199 10.1007/s11136-011-9960-1 PMC3323819

[19] Prinsen CAC Mokkink LB Bouter LM , et al. COSMIN Guideline for systematic reviews of patient-reported outcome measures. Qual Life Res 2018; 27: 1147–1157. 29435801 10.1007/s11136-018-1798-3 PMC5891568

[20] Fawcett AJL . Principles of assessment and outcome measurement for occupational therapists and physiotherapists: theory, skills and application. Chichester: J. Wiley, 2007.

[21] Tyson S Connell L . The psychometric properties and clinical utility of measures of walking and mobility in neurological conditions: a systematic review. Clin Rehabil 2009; 23: 1018–1033. 19786420 10.1177/0269215509339004

[22] Carmona C Sullivan JE Arceo R , et al. Development and preliminary validity study of a modified version of the upper extremity Fugl–Meyer assessment for use in telerehabilitation. J Neurol Phys Ther 2023; 47(4): 208–216. 37314323 10.1097/NPT.0000000000000447 PMC10487354

[23] Yu L Xiong D Guo L , et al. A remote quantitative Fugl–Meyer assessment framework for stroke patients based on wearable sensor networks. Comput Methods Programs Biomed 2016; 128: 100–110. 27040835 10.1016/j.cmpb.2016.02.012

[24] Liz L Da Silva TG Michaelsen SM . Validity, reliability, and measurement error of the remote Fugl–Meyer assessment by videoconferencing: tele-FMA. Phys Ther 2023; 103: 1–11. 10.1093/ptj/pzad054 37255324

[25] Hoffmann T Russell T Cooke H . Remote measurement via the internet of upper limb range of motion in people who have had a stroke. J Telemed Telecare 2007; 13: 401–405. 18078551 10.1258/135763307783064377

[26] Seo NJ Crocher V Spaho E , et al. Capturing upper limb gross motor categories using the Kinect® sensor. American Journal of Occupational Therapy 2019; 73: 1–10. 10.5014/ajot.2019.031682 PMC6638702 31318673

[27] MontJohnson A Cronce A Qiu Q , et al. Laboratory-based examination of the reliability and validity of kinematic measures of wrist and finger function collected by a telerehabilitation system in persons with chronic stroke. Sensors 2023; 23: 2656. 36904860 10.3390/s23052656 PMC10007090

[28] Park H-S Wu Y-N Ren Y , et al. A tele-assessment system for evaluating elbow spasticity in patients with neurological impairments. In: 2007 IEEE 10th International Conference on Rehabilitation Robotics. Noordwijk, Netherlands: IEEE, 2007, pp.917–922.

[29] Park H-S Peng Q Zhang L-Q . A portable telerehabilitation system for remote evaluations of impaired elbows in neurological disorders. IEEE Trans Neural Syst Rehabil Eng 2008; 16: 245–254. 18586603 10.1109/TNSRE.2008.920067

[30] Serradilla J Shi J Cheng Y , et al. Automatic assessment of upper limb function during play of the action video game, circus challenge: validity and sensitivity to change. In: 2014 IEEE 3rd International Conference on Serious Games and Applications for Health (SeGAH). Rio de Janeiro, Brazil: IEEE, 2014, pp.1–7.

[31] Uswatte G Hobbs Qadri L . A behavioral observation system for quantifying arm activity in daily life after stroke. Rehabil Psychol 2009; 54: 398–403. 19929121 10.1037/a0017501 PMC2799120

[32] Yang C Simpson LA Eng JJ . A pilot study for remote evaluation of upper extremity motor function after stroke: the arm capacity and movement test (ArmCAM). Am J Occup Ther 2023; 77: 7701205020. 36706274 10.5014/ajot.2023.050020

[33] Jordan HT Stinear CM . Accuracy and reliability of remote categorization of upper limb outcome after stroke. Neurorehabil Neural Repair 2024; 38: 167–175. 38357877 10.1177/15459683241231272 PMC10943605

[34] Bai J Song A . Development of a novel home based multi-scene upper limb rehabilitation training and evaluation system for post-stroke patients. IEEE Access 2019; 7: 9667–9677.

[35] Cai S Wei X Su E , et al. Online compensation detecting for real-time reduction of compensatory motions during reaching: a pilot study with stroke survivors. J NeuroEng Rehabil 2020; 17: 24–28. 32345335 10.1186/s12984-020-00687-1 PMC7189539

[36] Fan W Zhang Y Wang QM , et al. An interactive motion-tracking system for home-based assessing and training reach-to-target tasks in stroke survivors—a preliminary study. Med Biol Eng Comput 2020; 58: 1529–1547. 32405968 10.1007/s11517-020-02173-1

[37] Formstone L Pucek M Wilson S , et al. Myographic information enables hand function classification in automated Fugl–Meyer assessment. In: 2019 9th international IEEE/EMBS Conference on Neural Engineering (NER). San Francisco, CA, USA: IEEE, 2019, pp.239–242.

[38] Johnson MJ Feng X Johnson LM , et al. Potential of a suite of robot/computer-assisted motivating systems for personalized, home-based, stroke rehabilitation. J NeuroEngineering Rehabil 2007; 4: 6. 10.1186/1743-0003-4-6 PMC1821335 17331243

[39] Kim W-S Cho S Baek D , et al. Upper extremity functional evaluation by fugl-Meyer assessment scoring using depth-sensing camera in hemiplegic stroke patients. PLoS ONE 2016; 11: e0158640. 10.1371/journal.pone.0158640 PMC4930182 27367518

[40] Guo L Wang J Fang Q , et al. Motion recognition for unsupervised hand rehabilitation using support vector machine. In: 2012 IEEE Biomedical Circuits and Systems Conference (BioCAS). Hsinchu: IEEE, 2022, pp.104–107.

[41] Nguyen G Maclean J Stirling L . Quantification of compensatory torso motion in post-stroke patients using wearable inertial measurement units. IEEE Sensors J 2021; 21: 15349–15360.

[42] Rau C-L Chen Y-P Lai J-S , et al. Low-cost tele-assessment system for home-based evaluation of reaching ability following stroke. Telemedicine and e-Health 2013; 19: 973–978. 24138613 10.1089/tmj.2012.0300 PMC3850429

[43] Rodriguez-De-Pablo C Balasubramanian S Savic A , et al. Validating ArmAssist assessment as outcome measure in upper-limb post-stroke telerehabilitation. In: Proceedings of the Annual International Conference of the IEEE Engineering in Medicine and Biology Society, EMBS. Milan: IEEE, pp.4623–4626. 10.1109/EMBC.2015.7319424 26737324

[44] Song X Chen S Jia J , et al. Cellphone-based automated Fugl–Meyer assessment to evaluate upper extremity motor function after stroke. IEEE Transactions on Neural Systems and Rehabilitation Engineering : a publication of the IEEE Engineering in Medicine and Biology Society 2019; 27: 2186–2195. 31502981 10.1109/TNSRE.2019.2939587

[45] Venkataraman V Turaga P Lehrer N , et al. Decision support for stroke rehabilitation therapy via describable attribute-based decision trees. Conf Proc IEEE Eng Med Biol Soc 2014; 2014: 3154–3159. 10.1109/EMBC.2014.6944292 25570660

[46] Wang Y Yu L Fu J , et al. Remote intelligent Brunnstrom assessment system for upper limb rehabilitation for post-stroke based on extreme learning machine. Sheng Wu Yi Xue Gong Cheng Xue Za Zhi 2014; 31: 251–256. 25039122

[47] Wolf K Mayr A Nagiller M , et al. PoRi device: portable hand assessment and rehabilitation after stroke: poRi: ein transportables Gerät zum Einsatz im häuslichen Umfeld, fr die Bewertung und Rehabilitation der Hand nach einem Schlaganfall. at - Automatisierungstechnik 2022; 70: 1003–1017.

[48] Yang C Kerr A Stankovic V , et al. Human upper limb motion analysis for post-stroke impairment assessment using video analytics. IEEE Access 2016; 4: 650–659.

[49] Liu X Zhu Y Huo H , et al. Design of virtual guiding tasks with haptic feedback for assessing the wrist motor function of patients with upper motor neuron lesions. IEEE Trans Neural Syst Rehabil Eng 2019; 27: 984–994. 30969927 10.1109/TNSRE.2019.2909287

[50] Cai S Xie L . Automatic detection and reduction of compensation in stroke patients during robotic rehabilitation. In: 2023 9th International Conference on Control, Decision and Information Technologies (CoDIT). Rome, Italy: IEEE, 2023, pp.24–28.

[51] Casile A Fregna G Boarini V , et al. Quantitative comparison of hand kinematics measured with a markerless commercial head-mounted display and a marker-based motion capture system in stroke survivors. Sensors 2023; 23: 7906. 37765963 10.3390/s23187906 PMC10535006

[52] Dong Y Liu X Tang M , et al. Using virtual box and block test system as assessment of upper limb motor function in post-stroke patients: a feasibility study. Journal of Medical Biomechanics 2023; 38: 763–769.

[53] Garzo A Jung JH Arcas-Ruiz-Ruano J , et al. ArmAssist: a telerehabilitation solution for upper-limb rehabilitation at home. IEEE Robot Automat Mag 2023; 30: 62–71.

[54] Luo Z Lim TY . Development of a data-driven self-adaptive upper limb virtual rehabilitation system for post stroke elderly. In: 2023 IEEE Conference on Virtual Reality and 3D User Interfaces Abstracts and Workshops (VRW). Shanghai, China: IEEE, pp.635–636.

[55] Nayeem R Sohn WJ DiCarlo JA , et al. Novel platform for quantitative assessment of functional object interactions after stroke. IEEE Trans Neural Syst Rehabil Eng 2023; 31: 426–436. 36455078 10.1109/TNSRE.2022.3226067 PMC10079607

[56] Oubre B Lee SI . Detection and assessment of point-to-point movements during functional activities using deep learning and kinematic analyses of the stroke-affected wrist. IEEE J Biomed Health Inform 2023; 28(2): 1–9. 10.1109/JBHI.2023.3337156 38015679

[57] Zestas ON Souvatzis N Tselikas ND . A smart-glove approach in upper-limb rehabilitation assessment. In: 2024 Panhellenic Conference on Electronics & Telecommunications (PACET). Thessaloniki, Greece: IEEE, 2024, pp.1–4.

[58] Kim J Sin M Kim W-S , et al. Remote assessment of post-stroke elbow function using internet-based telerobotics: a proof-of-concept study. Front Neurol 2020; 11: 652–661. 33343489 10.3389/fneur.2020.583101 PMC7744560

[59] Lee WW Yen S-C Tay EBA , et al. A smartphone-centric system for the range of motion assessment in stroke patients. IEEE J Biomed Health Inform 2014; 18: 1839–1847. 25375681 10.1109/JBHI.2014.2301449

[60] Okita S De Lucena DS Chan V , et al. Measuring movement quality of the stroke-impaired upper extremity with a wearable sensor: toward a smoothness metric for home rehabilitation exercise programs. In: 2021 43rd Annual International Conference of the IEEE Engineering in Medicine & Biology Society (EMBC). Mexico: IEEE, 2021, pp.6691–6694. 10.1109/EMBC46164.2021.9629578 34892643

[61] Werner C Schönhammer JG Steitz MK , et al. Using wearable inertial sensors to estimate clinical scores of upper limb movement quality in stroke. Front Physiol 2022; 13: 877563. 35592035 10.3389/fphys.2022.877563 PMC9110656

[62] Geed S Grainger ML Mitchell A , et al. Concurrent validity of machine learning-classified functional upper extremity use from accelerometry in chronic stroke. Front Physiol 2023; 14: 1116878. 37035665 10.3389/fphys.2023.1116878 PMC10073694

[63] Zamin SA Tang K Stevens EA , et al. Abnormal motION capture in aCute stroke (BIONICS): a low-cost tele-evaluation tool for automated assessment of upper extremity function in stroke patients. Neurorehabil Neural Repair 2023; 37: 591–602. 37592867 10.1177/15459683231184186 PMC10602593

[64] Kim D-W Park JE Kim M-J , et al. Automatic assessment of upper extremity function and mobile application for self-administered stroke rehabilitation. IEEE Trans Neural Syst Rehabil Eng 2024; 32: 652–661. 38271165 10.1109/TNSRE.2024.3358497

[65] Guo CC Chiesa PA de Moor C , et al. Digital devices for assessing motor functions in mobility-impaired and healthy populations. Systematic Literature Review. J Med Internet Res 2022; 24: e37683. 10.2196/37683 PMC9723979 36409538

[66] Maceira-Elvira P Popa T Schmid A-C , et al. Wearable technology in stroke rehabilitation: towards improved diagnosis and treatment of upper-limb motor impairment. J NeuroEngineering Rehabil 2019; 16: 142. 10.1186/s12984-019-0612-y PMC6862815 31744553

[67] Torriani-Pasin C Demers M Polese JC , et al. Mhealth technologies used to capture walking and arm use behavior in adult stroke survivors: a scoping review beyond measurement properties. Disabil Rehabil 2021; 44(20): 1–13. 10.1080/09638288.2021.1953623 34297652

