# Supplementary material for: Remotely Assessing Motor Function and Activity of the Upper Extremity After Stroke: A Systematic Review of Validity and Clinical Utility of Tele-Assessments
Source: Clin Rehabil. 2024 Jun 5;38(9):1214–25. doi: 10.1177/02692155241258867 (PMC11487868; doi:10.1177/02692155241258867)
Supplement: sj-docx-1-cre-10.1177_02692155241258867 - Supplemental material for Remotely Assessing Motor Function and Activity of the Upper Extremity After Stroke: A Systematic Review of Validity and Clinical Utility of Tele-Assessments [file sj-docx-1-cre-10.1177_02692155241258867.docx]

# Supplementary Materials

**Online Only Supplement Table 1: Full Search Strategy for CINAHL**

**#1** (MH "Stroke+") OR TI ("cerebrovascular accident" OR "stroke" OR acute ischemic stroke" OR "transient ischemic attack" OR "brain infarction" OR "ischemi* attack" OR "ischemi* stroke" OR "hemorrhag* stroke" OR "brain* hemorrhag*" OR poststroke OR "post stroke" OR "post* infarct*" OR "cerebrovasc* accident" OR "cerebral infarct" OR apoplex* OR "cerebrovasc* diseas*" OR ((cerebrovasc* OR cerebral OR brain) N3 (accident OR lesion OR vasculopath* OR infarct*)) OR (hemipleg* OR hemipar* OR paresis* OR "stroke surviv*")) OR AB ("cerebrovascular accident" OR "stroke" OR acute ischemic stroke" OR "transient ischemic attack" OR "brain infarction" OR "ischemi* attack" OR "ischemi* stroke" OR "hemorrhag* stroke" OR "brain* hemorrhag*" OR poststroke OR "post stroke" OR "post* infarct*" OR "cerebrovasc* accident" OR "cerebral infarct" OR apoplex* OR "cerebrovasc* diseas*" OR ((cerebrovasc* OR cerebral OR brain) N3 (accident OR lesion OR vasculopath* OR infarct*)) OR (hemipleg* OR hemipar* OR paresis* OR "stroke surviv*"))

**#2** (MH "Task Performance and Analysis") OR (MH "Motor Skills+") OR (MH "Movement+") OR (MH "Psychomotor Performance+") (MH "Recovery+") OR (MH "Motor Activity") OR TI ("moto* funct*" OR "moto* dysfunct*” OR "sensorimotor* funct*" OR "sensorimotor*) OR AB ("moto* funct*" OR "moto* dysfunct*” OR "sensorimotor* funct*" OR "sensorimotor* OR moto*)) OR TI (task* N3 perform) OR AB (task* N3 perform) OR TI (motor* N3 (perform* OR funct* OR dysfunct* OR activit* OR assess* OR impair*)) OR AB (motor* N3 (perform* OR funct* OR dysfunct* OR activit* OR assess* OR impair*))

**#3** (MH "Disability Evaluation+") OR (MH "Remote Consultation") OR (MH "Videorecording+") AND ((TI (remote OR mobile OR app OR online OR digit* OR tele* OR distan* OR "tele*health*" OR "tele*therap*" OR "tele*rehab*" OR "mobile app*" OR telemedic* OR "e*health*" OR "m*health*" OR "tele*consult*" OR "tele*stroke*" OR videorecording OR "at a distanc*") OR AB (remote OR mobile OR app OR online OR digit* OR tele* OR distan* OR "tele*health*" OR "tele*therap*" OR "tele*rehab*" OR "mobile app*" OR telemedic* OR "e*health*" OR "m*health*" OR "tele*consult*" OR "tele*stroke*" OR videorecording OR "at a distanc*") OR (AB (assess* OR measur* OR detect* OR evaluat* OR reproduce*) N15 (remote OR mobile OR app OR online OR digit* OR tele* OR distan* OR "tele*health*" OR "tele*therap*" OR "tele*rehab*" OR "mobile app*" OR telemedic* OR "e*health*" OR "m*health*" OR "tele*consult*" OR "tele*stroke*" OR videorecording) OR TI (assess* OR measur* OR detect* OR evaluat* OR reproduce*) N15 (remote OR mobile OR app OR online OR digit* OR tele* OR distan* OR "tele*health*" OR "tele*therap*" OR "tele*rehab*" OR "mobile app*" OR telemedic* OR "e*health*" OR "m*health*" OR "tele*consult*" OR "tele*stroke*" OR videorecording)))

**#4** (MH "Upper Extremity+") OR (MH "Hand+") OR (MH "Hand Joints+") OR (MH "Shoulder Joint+") OR TI ("arm*" OR "hand*" OR "shoulder" OR "elbow*" OR "wrist*" OR "finger*" OR "joint* of should* region*" OR "forearm*") OR AB ("arm*" OR "hand*" OR "shoulder" OR "elbow*" OR "wrist*" OR "finger*" OR "joint* of should* region*" OR "forearm*") OR TI ("upp*" N3 ("limb*" OR "extrem*")) OR AB ("upp*" N3 ("limb*" OR "extrem*"))

**#5** #1AND #2 AND #3 AND #4

**Online Only Supplement Table 2:**
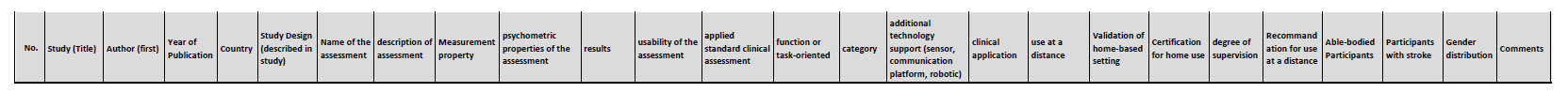
**Structured Form Data Extraction**

**Online Only Supplement Table 3: Risk of bias for the investigated tele-assessment for each study, following the COSMIN checklist (n=10)**

| **Name of the instrument** | **Study** | **Measurement properties** | | | | |
| --- | --- | --- | --- | --- | --- | --- |
|  |  | Reliability | Measurement Error | Criterion Validity | Hypotheses testing for construct validity* | Responsivness** |
| tUEFMA | Carmona et al. (2023) | Doubtful  + | Doubtful  ? | Very good  + |  |  |
| quantitative FMA framework | Yu et al. (2016) | Inadequate  - |  |  | Very good  + | Very good  ? |
| Tele-FMA | Liz et al. (2023) | Very good  + | Very good  + | Very good  + | Very good  + | Very good  + |
| Internet-based goniometer | Hoffmann et al. (2007) | Adequate  + | Very good  ? |  | Very good  ? | Very good  ? |
| computerized Mallet classification^a^ using Kinect sensor | Seo et al. (2019) | Doubtful  ? | Adequate  ? |  | Inadequate  ? |  |
| FOCUS-4 | Jordan & Stinear (2024) | Inadequate  ? |  |  |  |  |
| NJIT-HiVRS | MontJohnson et al. (2023) | Doubtful  -^a^ |  | Very good  - | Very good  ? |  |
| Portable Telerehabilitation System for Remote Evaluations of Impaired Elbows | Park et al. (2008) |  |  |  |  | Inadequate |
| CCAG | Serradilla et al. (2014) |  |  | Very good  + | Adequate  - | Adequate  - |
| FAABOS | Uswatte & Hobbs Qadri (2009) | Doubtful  + | Adequate  ? |  | Inadequate  ? | Inadequate  ? |
| ArmCAM | Yang et al. (2023) | Doubtful  + | Adequate  + | Very good  + | Very good  ? | Very good  ? |

*COSMIN item 9a: Comparison with other outcome measurement instruments (convergent validity)

**COSMIN item 10b: Construct Approach (i.e., hypotheses testing; comparison with other outcome measurement)

+ = sufficient rating; - insufficient rating; ? = Indeterminate rating

^a^No ICC was given for the entire assessment, but only for individual items. The rating was based on the lowest scores.

ArmCAM: Arm Capacity and Movement Test; CCAG: Circus Challenge Assessment Game; FAABOS: Functional Arm Activity Observation System; FOCUS-4: Fast Outcome Categorization of the Upper Limb after Stroke-4; NJIT-HiVRS: Kinematic Measures of Wrist and Finger Function by New Jersey Institute of Technology-Home Virtual Rehabilitation System; Tele-FMA: Tele-Fugl-Meyer Assessment for the Upper Extremity (excluding Lower Extremity); tUEFMA: Upper Extremety Fugl-Meyer Assessments for telerehabilitation.

**Online Only Supplement Table 4: Criteria for good measurement properties**

| Measurement property | Rating | Criteria |
| --- | --- | --- |
| Reliability | + | ICC or (weighted) kappa or Pearson/Spearman correlation ≥0.70 |
|  | ? | ICC or (weighted) kappa or Pearson/Spearman correlation not reported |
|  | - | ICC or (weighted) kappa or Pearson/Spearman correlation <0.70 |
| Measurement Error | + | SDC or LoA < MIC |
|  | ? | MIC not defined |
|  | - | SDC or LoA > MIC |
| Criterion Validity | + | Correlation with gold standard ≥ 0.70 OR AUC ≥ 0.70 |
|  | ? | Not all information for ‘+’ reported |
|  | - | Correlation with gold standard  <0.70 OR AUC<0.70 |
| Hypotheses testing for construct validity | + | ≥75% of the results is in accordance with predefined hypotheses |
|  | ? | No hypotheses defined (by the review team) |
|  | - | ≥75% of the results is not in accordance with predefined hypotheses |
| Responsiveness | + | ≥75% of the results is in accordance with predefined hypotheses OR AUC ≥0.70 |
|  | ? | No hypotheses defined (by the review team) |
|  | - | ≥75% of the results is not in accordance with predefined hypotheses OR AUC |

+ = sufficient rating; - insufficient rating; ? = Indeterminate rating

AUC = area under the curve, ICC = intraclass correlation coefficient, LoA = limits of agreement, MIC = minimal important change, SDC = smallest detectable change

**Online Only Supplement Table 5: Detailed Information on the Psychometric Properties of the included Tele-Assessments**

|  | **Validity** | | | | | | **Measurement Error** | | **Reliability** | | | | |
| --- | --- | --- | --- | --- | --- | --- | --- | --- | --- | --- | --- | --- | --- |
| Author (Year) | Convergent Validity | Criterion Validity | Construct Validity | Comparison | Statistics | Results | Statistics | Results | Intrarater- Reliability | Interrater-Reliability | Comparison | Statistics | Results |
| Carmona et al. (2023)^a^ | NR | 🗸 | NR | tUEFMA to FMA-UE | ICC  LoA | Total scores: 0.796, F= 8.46, CI: 0.568/0.91^1^  7.18 ± (1.96 × 5.2)  Subscale II: 0.648, F= 5.25, CI: 0.317/0.837  Subscale III : 0.819, F= 9.9, CI : 0.616/0.92  Subscale IV : 0.664, F= 4.86 , CI: 0.347/0.845  Subscale VII: 0.371 , F= 2.16, CI: −0.052/0.68 | NR | NR | NR | NR | NR | NR | NR |
| Yu et al. (2016) | NR | 🗸 | NR | quantitative Fugl-Meyer asssessment framework to FMA | ANOVA | Participant 1=0.613;  Participant 2=0.127;  Participant 3=0.482;  Participant 4=0.084;  Participant 5=0.581 | NR | NR | NR | NR | NR | NR | NR |
| Hoffmann et al. (2007) | NR | 🗸 | NR | Universal Goniometer | Correlation coefficients  LoA  (Mean absolute differences) | Affected arm  Shoulder flexion: >0.99 (2.1) LoA -5.0 to 3.2  Shoulder abduction: >0.99 (2.0), LoA -4.8 to 5.4  Elbow flexion: 0.99 (1.1), LoA -2.5 to 2.7  Forearm supination: >0.99 (1.6), LoA -1.5 to 4.5  Forearm pronation: 0.97 (1.8). LoA-4.0 to 4.4  Wrist flexion: >0.99 (1.7),,LoA -3.9 to 5.1  Wrist extension: >0.99 (2.0), LoA-1.5 to 5.1 | NR | NR | 🗸 | 🗸 | Universal Goniometer | ICC  (Mean absolute differences) | Intrarater Reliability for Affected Arm: >0.99 (1.1)  Shoulder flexion: >0.99 (1.1)  Shoulder abduction: >0.99 (1.9)  Elbow flexion: 0.99 (1.2)  Forearm supination: >0.99 (1.1)  Forearm pronation: 0.98 (2.0)  Wrist flexion: >0.99 (1.1)  Wrist extension: >0.99 (1.5)  Interrater Reliability for Affected Arm:  Shoulder flexion: >0.99 (2.1)  Shoulder abduction: >0.99 (2.1)  Elbow flexion: 0.98 (2.1)  Forearm supination: >0.99 (2.0)  Forearm pronation: 0.99 (1.9)  Wrist flexion: 0.99 (3.0)  Wrist extension: 0.99 (2.5) |
| Liz et al. (2023)^c^ | 🗸 | 🗸 | 🗸 | I^2^: SIS  II^3^: FMA | I and II: ICC, LoA | I: ρ =0.82 (95% CI 0.43-0.95); p=0.002  II: FMA-UE: ρ=0.99 (95%CI 0.96-0.99); p<0.0001, SIS hand function: ρ=0.93 (95%CI 0.73-0.98); p<0.0001] | SEM, MDC | SEM: 0.9-2.2 points  MDC: <=2.5 points | 🗸 | 🗸 | None (scoring by recorded videos) | ICC  Weighted Kappa | Intrarater Reliability: for Tele-FMA-UE: 0.99; 95% CI=0.99-1.00  Interrater Reliability for Tele-FMA-UE: 0.99, 95% CI=0.98-1,00^4^  FMA-UE individual items that need resistance im-person vs Tele-FMA:  Wrist stable. Elbow at 90° (0-2): 1.00 (1.00-1.00)  Wrist stable. Elbow extended (0-2): 0.91 (0.74-1.00)  Hook grasp (0-2): 1.00 (1.00-1.00)  Lateral prehension (0-2): 1.00 (1.00-1.00)  Opposition thumb and index (0-2): 1.00 (1.00-1.00)  Cylindrical grasp (0-2): 1.00 (1.00-1.00)  Spherical grasp (0-2): 1.00 (1.00-1.00) |
| Seo et al. (2019) |  | NR | NR | NR | NR | NR | NR | NR | NR | 🗸 | Computerized Mallet classification to collaborative visual assessment using video recordings | Cohen’s K coefficient | For all tasks: 0.66 for Mallet assessment,  Agreement: 77%  Agreement Task 1: 100%  Agreement Task 2: 86%  Agreement Task 3: 71%  Agreement Task 4: 43%  Agreement Task 5: 86% |
| Jordan & Stinear (2024) |  | NR | NR | NR | NR | NR | NR | NR | 🗸 | NR | First to second remote assessment | NR | Correct classification of 82% of the participants |
| MontJohnson et al. (2023) |  | NR^b^ | NR | NR | NR | NR | NR | NR | 🗸 | NR | In-person to remote  and remote to remote | ICC | In-person to remote:  HOR: 0.857, CI 0.381–0.973  HOA: 0.910, CI 0.496–0.987  WER: 0.883, CI 0.472–0.979  WEA: 0.581, CI (-) 0.309–0.928  PSR: 0.911, CI 0.577–0.984  PSA: 0.969, CI 0.831–0.995  Remote to remote:  HOR: 0.824, CI 0.280–0.967  HOA: 0.794, CI 0.098–0.968  WER: 0.905, CI 0.549–0.983  WEA: 0.676, CI (-) 0.161–0.947  PSR: 0.967, CI 0.820–0.994  PSA: 0.785, CI 0.176–0.959 |
| Park et al. (2008) | NR | 🗸 | NR | Manually measurements, affixed to master device | Correlation, MPD | Passive ROM Extension ρ =1.000, MPD: 9.4%  Passive ROM Flexion ρ = 0.990, MPD: 2.9%  Active ROM Extension ρ=0.992, MPD: 7.3%  Active ROM Flexion ρ=0.961, MPD 1.5% | NR | NR | NR | NR | NR | NR | NR |
| Park et al. (2007) | **No results for psychometric properties in this study**  **-> no data extraction** | | | |  |  |  |  |  |  |  |  |  |
| Serradilla et al. (2014) | 🗸 |  |  | CAHAI-9 | Correlation^8^  ROC analysis | 0.998^9^  r=0.54 (overall)^10^  chronic group r=0.33^10^  acute group r=0.63^10^  ROC analysis: p=.50 | NR | NR | NR | NR | NR | NR | NR |
| Uswatte & Hobbs Qadri (2009) | 🗸 | NR | NR | Average ratings for each 15-minute video segment; more-impaired to less-impaired arm | Pearson  Correlation | r(14) = .55 (p<.05) | NR | NR | NR | 🗸 | Between pairs of observers | Cohen’s K coefficient | Κ = .84 (SD = .09) |
| Yang et al. (2023) | NR | 🗸 | 🗸 | REACH,  SIS-Hand  FMA-UE  ARAT | Pearson and Spearman  Correlation | REACH with ArmCAM: .870 (0.715, 0.943)^5^  SIS -Hand with ARMCAM .811 (0.641, 0.905)^6^  REACH with ARMCAM .870 (0.715, 0.943)^6^  FMA-UE with ARMCAM: .944 (0.708, 0.990)^6,7^  ARAT with ARMCAM: .936 (0.673, 0.989)^6,7^ | NR | NR | 🗸 | 🗸 | Two raters, by video observation | ICC  Cohen’s K  SEM  MDC | ICC intra-rater reliability: .997  ICC inter-rater reliability: .993  SEM: 0.74 points  MSC: 2.05 points |

ANOVA: Analysis of variance; CAHAI-9: Chedoke Arm and Hand Assessment Inventory; CI: Confidence Interval; FMA: Fugl-Meyer Assessment; HOA: Hand Opening Accuracy; HOR: Hand Opening Range; ICC: Intraclass Correlation Coefficient; LoA: Limits of Agreement; MDC: Minimal detectable Change; MPD Maximum Percentage Difference between in-person and tele-assessment; NR: not reported; PSA: Pronation-Supination Accuracy; PSR: Pronation-Supination Range; REACH: Rating of Everyday Arm-use in the Community and Home scale; ROC: receiver operating characteristic; ROM: Range of Motion; SEM: Standard Error of Measurement,; SIS: Stroke Impact Scale; UE: Upper Extremity; WEA: Wrist Extension Accuracy; WER: Wrist Extension Range.

^a^adaption of the original instrument: subscales I and V (reflexes), VI (wrist) and VIII (Coordination/Speed) removed. Modifications in subscales III (Active Movement Mixing Synergies) and VII (Hand). Total max. score for tUEFMA: 44.

^b^Validity was assessed in the second study of this paper. However, because Study 2 was not conducted at a distance, the results of this part are not considered in the review.

^c^adaption of the original instrument: reflex items were not assessed, resistance provided by caregiver where necessary.

^1^exception: poor agreement for subscale VII (Hand)

^2^I: Comparison, statistics, and results for convergent validity

^3^II: Comparison, statistics, and results for construct validity

^4^ All the individual items presented excellent intrarater reliability (κw≥0.70), except for two items (κw<0.70), namely: item 1.1 – Flexor Synergy– shoulder retraction (κw=0.63; 95%CI=0.42 - 0.83) and item 1.4 – Flexor Synergy – external shoulder rotation (κw=0.66; 95%CI=0.47-0.85).

^5^Pearson correlation coefficient

^6^Spearman rank correlation coefficient

^7^Subgroup analysis with 10 participants

^8^Additionally a cross-sectional validity (using between-subjects correlation) and longitudinal validity (within-subjects correlation in groups) was performed.

^9^Results Cross-sectional validity

^10^Results Longitudinal validity

**Online Only Supplement Table 6: Detailed Description Clinical Utility**

| **Instrument Name** | **Technical Equipment** | **Cost** | **Time** | **Energy and Effort** | **Portability** | **Acceptability** |
| --- | --- | --- | --- | --- | --- | --- |
| **tUEFMA** | - Phone/computer, internet, camera, Zoom, WhatsApp or Facetime | Requires only basic telecommunication technology, no additional software/  hardware required. | NR | At the beginning of each remote session, privacy-, and safety concerns (such as positioning and fall risks) were discussed. Additionally, adjustments to the camera position were made to best optimize the capture of the triplanar arm motions. During the session, equipment modifications were made for certain participants to enhance arm visualization.  Participants used personal devices,  including desktop computers, laptops, tablets, smartphones,  and webcams. | - Given the equipment mentioned, it appears to be easily portable. | NR |
| **quantitative Fugl-Meyer asssessment framework** | - 2 accelerometer sensors, seven flex sensors that are integrated into a glove. - Transmission of data to the computer through ZigBee protocol. - web server database - Website and APP (Android or iOS) to view training record and Fugl-Meyer Score - Home setting: desktop or laptop computer, connection to Internet, one camera and remote videoconferencing software - Remote Rehabilitation Training and Assessment Software (RRTAS): data sampling and management | NR | NR | Participants wear sensors, while being guided by physicians and technical support engineers through remote video conferencing software.  Flex sensors are integrated into a glove to make them easy for patients to wear.  For the experiments in home settings, it is required that the participants have a desktop or laptop and a stable Internet connection. | - Given the equipment mentioned, it appears to be easily portable. | It is required that the participants have access to a desktop or laptop computer in their households, with an internet connection properly configured.  Among the 24 participants, only 7 patients fulfilled the above requirements. Additionally, due to the lack of awareness of the importance of rehabilitation, another 2 patients did not agree to the continued rehabilitation at home.  Hence, only 5 out of the 24 participants were involved in the experiments in the home settings conducted at the home settings. |
| **Tele-FMA** | - WhatsApp videoconferencing, camera, phone, or computer | NR | Length of time taken to apply Tele-FMA did not differ from time taken to apply the in-person FMA.  Duration of the total Tele-FMA was on average 34.12 (SD 11.46) min. For the TeleFMA-UE it was 21.07 (SD 08.51) min and for the Tele-FMA-LE, 13.05 (SD 05.21) min. | Before the remote assessment via videoconferencing took place, a document was sent to the participant and caregiver explaining how to arrange the assessment environment, position the camera and prepare the necessary materials.  Materials that are additionally needed are readily available or present in the household (e.g., pen, 500ml bottle, armless chair without wheels).  Patients with severe impairments (e.g., low trunk control) may find it challenging to undergo Tele-FMA, as caregivers may struggle to position both the device and the patient optimally. | - Given the equipment mentioned, it appears to be easily portable. | Patient's perceptions were collected via semi-structured interviews:  1) Issues with internet signal during the assessment (n=9)  2) difficulty understanding the correct way to execute the movements (n=8)  3) had to reschedule their assessments due to problems with the internet signal (n=3)  4) 59% needed presence of a caregiver most of the time because they felt their limitations hindered them from using the instrument.  Perceived advantages: Approximately 60% of participants identified the primary benefit as being able to complete the assessment without leaving their homes. Perceived disadvantage: 32% mentioned the physical absence of the therapist during the assessment, and the consequent lack of physical touch and guidance when attempting to execute the movements. However, a considerable percentage (41%) of the participants reported experiencing no disadvantage with the Tele-FMA. |
| **Internet-based goniometer** | - Telerehabilitation system based on Russell et al. (2003) | NR | NR | Some participants had trouble maintaining required positions. Assessor had to take the measurements as quickly as possible which may have inflated the measurement error.  Participants were seated in a chair for all measurements and the assessor demonstrated the desired movement to the participant prior to each measure | - Given the equipment mentioned, it appears to be easily portable. | NR |
| **computerized Mallet classification** | - Kinect sensor (Microsoft Corp., Redmond, WA) - Computerized assessment program OpenNI Software Development Kit, custom-developed C++ program | Kinect is low cost | NR | No other additional objects are required, so it can be assessed using the Kinect. However, object grasping, and manipulation cannot be assessed using Kinect unless additional sensors are used on patient's body or objects | - Given the equipment mentioned, it appears to be easily portable. | NR |
| **FOCUS-4** | - Videoconferencing Tool | NR | Approx. 5 minutes | Items required to perform the assessment can be send to participants (e.g., Cardboard boxes filled with sand, marble and 2 jar lids).  Caregiver or family member needs to be present to hold the phone.  Instructions to participants were given during the videocall. | - Given the equipment mentioned, it appears to be easily portable. | NR |
| **NJIT-HiVRS** | - Leap Motion Controller (LMC): a pair of cameras and a set of infrared LEDs - Arm support - Laptop - Video conferencing camera | NR | NR | NR | - Given the equipment mentioned, it appears to be easily portable. | System Usability Scale Testing:  They conducted the System Usability Scale with 10 items. (For study I): questions like “I think that I would like to use this system frequently”). Score 0 to 100 (higher score = higher levels of perceived usability, score of 68 or more SUS means device is "acceptable to use".)  Therapist's SUS scores: Scores ranged from 70 to 90. Mean was 83.1 (SD =6.4)  Patient's SUS scores: ranged from 70 to 85. Mean was 80.4 (SD = 5.2) |
| **Portable Telerehabilit-ation System for Remote Evaluations of Impaired Elbows** | - Haptic Device with a Mannequin Arm (Master) - Portable Stretching Device (Slave) - WebCams with Microphone - Laptops or PC - Internet-Connection - Program for videoconferencing | Prototyped master and slave used commonly available materials.  Authors suggested a low-cost leasing option. | NR | The motor in the portable stretching device had low power requirements, enabling allowing potential use of batteries, servomotor max torque of 7 Nm.  Tool could be used even without an online connection, functioning as portable limb stretching device, thereby → measuring biomechanical properties | - Slave device is portable: Size 14x11x19cm3, Weight 2.2kg; master Dimensions: 16x20x25cm3, Weight 4kg. - Given the equipment mentioned, it appears to be easily portable. | NR |
| **CCAG** | - Game Circus Challenge (produced for company Limbs Alive, video game studio Pitbull) - One state-of-the-art prototype wireless controller - Laptop | Low cost, standard hardware | Time requirement to complete the assessment does not exceed 20 minutes. | Patients were introduced to CCAG at baseline and asked to play the game in their home each day for 30 minutes. | - Only the game, a wireless controller and laptop are required. - Given the equipment mentioned, it appears to be easily portable. | NR |
| **FAABOS** | - Accelerometry system - Miniature, wide-angle video camera, motion-triggered recorder | NR | NR | NR | - Given the equipment mentioned, it appears to be easily portable. | NR |
| **ArmCAM** | - Computer, tablet, or phone with a webcam - Videoconferencing software (Zoom Video) | NR | It takes 15min to administer the assessment. | Easy to use, only requires items that can be found in home.  Participants used tablets and phones.  They often experienced difficulties in positioning their devices and adjusting the camera angle. | - Given this equipment, it appears to be easily portable. | NR |
|  |  |  |  |  |  |  |

ArmCAM: Arm Capacity and Movement Test; CCAG: Circus Challenge Assessment Game; FAABOS: Functional Arm Activity Observation System; FMA: Fugl-Meyer Assessment; FOCUS-4: Fast Outcome Categorization of the Upper Limb after Stroke-4; NJIT-HiVRS: Kinematic Measures of Wrist and Finger Function by New Jersey Institute of Technology-Home Virtual Rehabilitation System; NR: Not reported; SD: Standard Deviation; Tele-FMA: Tele-Fugl-Meyer Assessment for the Upper Extremity (excluding Lower Extremity); tUEFMA: Upper Extremeity Fugl-Meyer Assessments for telerehabilitation.
