# Supplementary material for: Remotely Assessing Motor Function and Activity of the Upper Extremity After Stroke: A Systematic Review of Validity and Clinical Utility of Tele-Assessments
Source: Clin Rehabil. 2024 Jun 5;38(9):1214–25. doi: 10.1177/02692155241258867 (PMC11487868; doi:10.1177/02692155241258867)
Supplement: sj-pdf-2-cre-10.1177_02692155241258867 - Supplemental material for Remotely Assessing Motor Function and Activity of the Upper Extremity After Stroke: A Systematic Review of Validity and Clinical Utility of Tele-Assessments [file sj-pdf-2-cre-10.1177_02692155241258867.pdf]

Box 6. Reliability

Design requirements	**very good**	**adequate**	**doubtful**	**inadequate**	**NA**
1 Were patients stable in the interim period on the construct to be measured?	Evidence provided that patients were stable	Assumable that patients were stable	Unclear if patients were stable	Patients were NOT stable	
2 Was the time interval appropriate?	Time interval appropriate		Doubtful whether time interval was appropriate or time interval was not stated	Time interval NOT appropriate	
3 Were the test conditions similar for the measurements? e.g. type of administration, environment, instructions	Test conditions were similar (evidence provided)	Assumable that test conditions were similar	Unclear if test conditions were similar	Test conditions were NOT similar	
Statistical methods	**very good**	**adequate**	**doubtful**	**inadequate**	**NA**
4 For continuous scores: Was an intraclass correlation coefficient (ICC) calculated?	ICC calculated and model or formula of the ICC is described	ICC calculated but model or formula of the ICC not described or not optimal. Pearson or Spearman correlation coefficient calculated with evidence provided that no systematic change has occurred	Pearson or Spearman correlation coefficient calculated WITHOUT evidence provided that no systematic change has occurred or WITH evidence that systematic change has occurred	No ICC or Pearson or Spearman correlations calculated	Na
5 For dichotomous/nominal/ ordinal scores: Was kappa calculated?	Kappa calculated			No kappa calculated	Na
6 For ordinal scores: Was a weighted kappa calculated?	Weighted Kappa calculated		Unweighted Kappa calculated or not described		Na
7 For ordinal scores: Was the weighting scheme described? e.g. linear, quadratic	Weighting scheme described	Weighting scheme NOT described			Na
Other					
8 Were there any other important flaws in the design or statistical methods of the study?	No other important methodological flaws		Other minor methodological flaws	Other important methodological flaws	

Box 7. Measurement error

Design requirements	**very good**	**adequate**	**doubtful**	**Inadequate**	**NA**
1 Were patients stable in the interim period on the construct to be measured?	Patients were stable (evidence provided)	Assumable that patients were stable	Unclear if patients were stable	Patients were NOT stable	
2 Was the time interval appropriate?	Time interval appropriate		Doubtful whether time	Time interval NOT	

			interval was appropriate or time interval was not stated	appropriate	
3 Were the test conditions similar for the measurements? (e.g. type of administration, environment, instructions)	Test conditions were similar (evidence provided)	Assumable that test conditions were similar	Unclear if test conditions were similar	Test conditions were NOT similar	
Statistical methods	**very good**	**adequate**	**doubtful**	**Inadequate**	**NA**
4 For continuous scores: Was the Standard Error of Measurement (SEM), Smallest Detectable Change (SDC) or Limits of Agreement (LoA) calculated?	SEM, SDC, or LoA calculated	Possible to calculate LoA from the data presented		SEM calculated based on Cronbach's alpha, or on SD from another population	Not applicable
5 For dichotomous/ nominal/ ordinal scores: Was the percentage (positive and negative) agreement calculated?	% positive and negative agreement calculated	% agreement calculated		% agreement not calculated	Not applicable
Other					
Were there any other important flaws in the design or statistical methods of the study?	No other important methodological flaws		Other minor methodological flaws	Other important methodological flaws	

Box 8. Criterion validity

Statistical methods	**very good**	**adequate**	**doubtful**	**inadequate**	**NA**
1 For continuous scores: Were correlations, or the area under the receiver operating curve calculated?	Correlations or AUC calculated			Correlations or AUC NOT calculated	Na
2 For dichotomous scores: Were sensitivity and specificity determined?	Sensitivity and specificity calculated			Sensitivity and specificity NOT calculated	Na
Other					
3 Were there any other important flaws in the design or statistical methods of the study?	No other important methodological flaws		Other minor methodological flaws	Other important methodological flaws	

Box 9. Hypotheses testing for construct validity

9a. Comparison with other outcome measurement instruments (convergent validity)

Design requirements	**very good**	**adequate**	**doubtful**	**inadequate**	**NA**
1 Is it clear what the comparator instrument(s) measure(s)?	Constructs measured by the comparator instrument(s) is clear			Constructs measured by the comparator instrument(s) is not clear	
2 Were the measurement properties of the comparator instrument(s) sufficient?	Sufficient measurement properties of the comparator instrument(s) in a population	Sufficient measurement properties of the comparator	Some information on measurement properties of the comparator instrument(s) in	No information on the measurement properties of the comparator instrument(s), OR evidence of	

	similar to the study population	instrument(s) but not sure if these apply to the study population	any study population	insufficient measurement properties of the comparator instrument(s)	
Statistical methods					
3 Were design and statistical methods adequate for the hypotheses to be tested?	Statistical methods applied appropriate	Assumable that statistical methods were appropriate	Statistical methods applied NOT optimal	Statistical methods applied NOT appropriate	
Other					
4 Were there any other important flaws in the design or statistical methods of the study?	No other important methodological flaws		Other minor methodological flaws (e.g. only data presented on a comparison with an instrument that measures another construct)	Other important methodological flaws	

10b. Construct approach (i.e. hypotheses testing; comparison with other outcome measurement instruments)

Design requirements	**very good**	**adequate**	**doubtful**	**inadequate**	**NA**
4 Is it clear what the comparator instrument(s) measure(s)?	Constructs measured by the comparator instrument(s) is clear			Constructs measured by the comparator instrument(s)	
5 Were the measurement properties of the comparator instrument(s) sufficient?	Sufficient measurement properties of the comparator instrument(s) in a population similar to the study population	Sufficient measurement properties of the comparator instrument(s) but not sure if these apply to the study population	Some information on measurement properties of the comparator instrument(s) in any study population	NO information on the measurement properties of the comparator instrument(s) OR evidence of insufficient quality of comparator instrument(s)	5 Were the measurement properties of the comparator instrument(s) sufficient?
Statistical methods					
6 Were design and statistical methods adequate for the hypotheses to be tested?	Statistical methods applied appropriate	Assumable that statistical methods were appropriate	Statistical methods applied NOT optimal	Statistical methods applied NOT appropriate	
Other					
7 Were there any other important flaws in the design or statistical methods of the study?	No other important methodological flaws		Other minor methodological flaws	Other important methodological flaws	

[1] If the scale is not based on a reflective model, internal consistency is not relevant
